# Supplementary material for: Femora from an exceptionally large population of coeval ornithomimosaurs yield evidence of sexual dimorphism in extinct theropod dinosaurs
Source: eLife. 2023 Jun 13;12:e83413. doi: 10.7554/eLife.83413 (PMC10264075; doi:10.7554/eLife.83413)
Supplement: Supplementary file 1. [file elife-83413-supp1.docx]

Supplementary File 1: Statistical parameters used in this study for size-effect and cluster attribution

| Parameters | Complete femora | Distal epiphyses |
| --- | --- | --- |
| Log centroid size vs. PC1 scores | *r²*: 0.12; *p-value* > 0.1 | *r²*: 0.07 ; *p-value* > 0.1 |
| Model selected by the EM | univariate, equal variance | univariate, equal variance |
| Number of components | 2 | 2 |
| BIC | 46.54 | 48.47 |
| Log-likelihood | 27.87 | 30.12 |
| Mixing probabilities for each cluster | 0.61; 0.39 | 0.52; 0.48 |
| Highest uncertainty for cluster attribution/specimen | 0.0001 | 0.02 |
