# Supplementary material for: Femora from an exceptionally large population of coeval ornithomimosaurs yield evidence of sexual dimorphism in extinct theropod dinosaurs
Source: eLife. 2023 Jun 13;12:e83413. doi: 10.7554/eLife.83413 (PMC10264075; doi:10.7554/eLife.83413)
Supplement: Supplementary file 2. [file elife-83413-supp2.docx]

Supplementary File 2: Cluster attribution for complete femora studied in analyses for both complete femora and distal epiphyses

| Specimen | Morph attribution for complete femora | Morph attribution for distal epiphyses |
| --- | --- | --- |
| ANG 10 84 | A | A |
| ANG 10 90 | B | B |
| ANG 11 1271 | B | B |
| ANG 13 2780 | A | A |
| ANG 14 R392 | B |  |
| ANG 15 3865 | A | A |
| ANG 15 4182 | A |  |
| ANG 16 5017 | A |  |
| ANG 16 5140 | A |  |
| ANG 16 5120 | B | B |
