# Supplementary material for: Femora from an exceptionally large population of coeval ornithomimosaurs yield evidence of sexual dimorphism in extinct theropod dinosaurs
Source: eLife. 2023 Jun 13;12:e83413. doi: 10.7554/eLife.83413 (PMC10264075; doi:10.7554/eLife.83413)
Supplement: Supplementary file 3. — * refers to specimens digitized with the NextEngine, other specimens were digitized using the Artec EVA. Abbreviations: Col. Nb., collection number; F.L., femoral length (maximal distance between proximal and distal epiphyses); L, left; P.W., proximal width; R, right. Specimens are available online on MorphoSource at https://www.morphosource.org/projects/000519447?locale=en. [file elife-83413-supp3.docx]

Supplementary File 3: Specimens used in this study. * refers to specimens digitized with the NextEngine, other specimens were digitized using the Artec EVA. Abbreviations: Col. Nb., collection number; F.L., Femoral length (maximal distance between proximal and distal epiphyses); L, left; P.W. proximal width; R, right.

| Col. Nb. | Bone | Integrity | Side | P.W. (mm) | D.W. (mm) | F.L. (mm) | Morph |
| --- | --- | --- | --- | --- | --- | --- | --- |
| ANG 10 43 | Femur | Proximal | L | 59 |  |  |  |
| ANG 10 53 | Femur | Proximal | R | 66 |  |  |  |
| ANG 10 84 | Femur | Complete | R | 66 | 62 | 379.7 | A |
| ANG 10 86 | Femur | Proximal | L | 40 |  |  |  |
| ANG 10 90 | Femur | Complete | L | 60 | 71 | 394.8 | B |
| ANG 10 171 | Femur | Distal | L | 86 | 82 | 491 | B |
| ANG 11 735 | Femur | Distal | R |  | 51 |  | A |
| ANG 11 811a | Femur | Proximal | R | 67 |  |  |  |
| ANG 11 811b | Femur | Distal | R |  | 67 |  | B |
| ANG 11 1107 | Femur | Distal | R |  | 64 |  | B |
| ANG 11 1209 | Femur | Proximal | L | 79 |  |  |  |
| ANG 11 1271 | Femur | Complete | R | 73 | 78 | 384.8 | B |
| ANG 12 1844 | Femur | Distal | L |  | 70 |  | B |
| ANG 13 2282 | Femur | Proximal | L | 71 |  |  |  |
| ANG 13 2381 | Femur | Proximal | R | 78 |  |  |  |
| ANG 13 2428 | Femur | Distal | L |  | 70 |  | A |
| ANG 13 2451 | Femur | Distal | R |  | 60 |  | A |
| ANG 13 2749 | Femur | Proximal | L | 77 |  |  |  |
| ANG 13 2757 | Femur | Proximal | R | 43 |  |  |  |
| ANG 13 2780 | Femur | Complete | L | 58 | 52 | 319.7 | A |
| ANG 13 2807 | Femur | Distal | R |  | 78 |  | A |
| ANG 14 R392 | Femur | Complete | R | 74 | 72 | 381.9 | B |
| ANG 14 3188 | Femur | Distal | L |  | 66 |  | A |
| ANG 14 3488 | Femur | Proximal | L | 82 |  |  |  |
| ANG 14 3516 | Femur | Proximal | R | 45 |  |  |  |
| ANG 14 3570 | Femur | Proximal | R | 83 |  |  |  |
| ANG 15 3865 | Femur | Complete | R | 72 | 65 | 388.8 | A |
| ANG 15 4182 | Femur | Complete | L | 51 | 51 | 302.9 | A |
| ANG 16 5017 | Femur | Complete | L | 68 | 67 | 390.9 |  |
| ANG 16 5106 | Femur | Proximal | R | 44 |  |  |  |
| ANG 16 5120 | Femur | Complete | R | 65 | 64 |  | B |
| ANG 16 5140 | Femur | Complete | R | 64 | 63 | 364.7 | A |
| ANG 16 5077 | Femur | Distal | R |  | 51 |  | A |
| ANG 16 5120 | Femur | Complete | R | 65 | 64 | 356.3 |  |
| ANG 17 5704 | Femur | Proximal | L | 64 |  |  |  |
| ANG 17 5709 | Femur | Distal | L |  | 63 |  | B |
| ANG 19 6825* | Femur | Distal | L |  |  |  | A |
| ANG 20 7346* | Femur | Distal | R |  | 99 |  | B |
