# Supplementary material for: Femora from an exceptionally large population of coeval ornithomimosaurs yield evidence of sexual dimorphism in extinct theropod dinosaurs
Source: eLife. 2023 Jun 13;12:e83413. doi: 10.7554/eLife.83413 (PMC10264075; doi:10.7554/eLife.83413)
Supplement: Supplementary file 4. — Abbreviations: s, anatomical landmarks; c, sliding semilandmarks on curves. [file elife-83413-supp4.docx]

Supplementary File 4: Landmark scheme of the femur according to the numerotation shown in Figure S4. Abbreviations: s, anatomical landmarks; c, sliding semilandmarks on curves.

| **N.** | **Description** |
| --- | --- |
| 0 | Most distal point of the fovea |
| 1 | Most anterior point of the ALT |
| 2 | Maximum of concavity on the lateral part of the ALT |
| 3 | Intersection between the most proximal point of the fovea and the lateral border of the AMT |
| 4 | Most posterior point of the proximal border of the greater trochanter |
| 5 | Most anterior point of the proximal border of the greater trochanter |
| 6 | Most distal point of the anterior border of the greater trochanter |
| 7 | Most posterior point of the proximal border of the lesser trochanter (anterior trochanter) |
| 8 | Most distal point of the anterior border of the lesser trochanter (accessory trochanter) |
| 9 | Foramen in the depression between the lesser trochanter and the femoral head |
| 10 | Most proximal point of the 4^th^ trochanter |
| 11 | Maximum of concavity of the distal part of the 4^th^ trochanter |
| 12 | Most distal part of the 4^th^ trochanter |
| 13 | Most proximal point of the CFL-BR fossa |
| 14 | Most distal point of the CFL-BR fossa |
| 15 | Most proximal point of the ectocondylar tuberosity |
| 16 | Intersection between the most distal part of the MDC (mediodistal crest) and the most proximal part of the MF (medial flange) |
| 17 | Most posterior point of the MF |
| 18 | Maximum of concavity on the most proximal point of the medial condyle |
| 19 | Maximum of concavity between the medial condyle and the posterior intercondylar fossa |
| 20 | Maximum of concavity between the crista tibiofibularis and the posterior intercondylar fossa |
| 21 | Maximum of concavity on the most proximal point of the crista tibiofibularis |
| 22 | Maximum of concavity between the crista tibiofibularis and the lateral condyle |
| 23 | Maximum of concavity in the anterior intercondylar fossa |
| 24 | Maximum of concavity on the most distal surface of the distal epiphyses. |
| c0; c1 | Medial border of the fovea |
| c2; c3 | Proximal and anterior border of the greater trochanter |
| c4; c5 | Proximal and anterior border of the lesser trochanter |
| c6; c7 | Outline of the CFL-BR fossa |
| c8; c9 | Posterior border of the 4^th^ trochanter |
| c10; c13 | Outline of the medial flange |
| c14 c15 | Outline of the distal border of the lateral condyle |
