# Supplementary material for: Femora from an exceptionally large population of coeval ornithomimosaurs yield evidence of sexual dimorphism in extinct theropod dinosaurs
Source: eLife. 2023 Jun 13;12:e83413. doi: 10.7554/eLife.83413 (PMC10264075; doi:10.7554/eLife.83413)
Supplement: Supplementary file 5. — Abbreviations: s, anatomical landmarks; c, sliding semilandmarks on curves. [file elife-83413-supp5.docx]

Supplementary File 5: Landmark scheme of the tibia according to the numerotation shown in Figure S5. Abbreviations: s, anatomical landmarks; c, sliding semilandmarks on curves.

| **N.** | **Description** |
| --- | --- |
| 0 | Most proximal point of the maximum of concavity in the intercondylar groove on the tibial head |
| 1 | Most proximal point of the medial side of the cnemial crest |
| 2 | Maximum of concavity along the distal part of medial side of the cnemial crest |
| 3 | Most distal point of the lateral condyle |
| 4 | Most anterior point of the distal border of the lateral condyle |
| 5 | Most posterior point of the proximal border of the lateral side of the cnemial crest |
| 6 | Most anterior point of the proximal border of the lateral side of the cnemial crest |
| 7 | Most anterior point of the anterior border of the lateral side of the cnemial crest |
| 8 | Most posterior point of along the anterior border of the lateral side of the cnemial crest |
| 9 | Most distal point of the anterior border of the lateral side of the cnemial crest |
| 10 | Most proximal point of the fibular crest |
| 11 | Maximum of concavity of the distal part of the fibular crest |
| 12 | Most distal point of the fibular crest |
| 13 | Foramen on the posterior side of the fibular crest |
| 14 | Most distal point of the surface of contact with the fibula |
| 15 | Maximum of concavity on the proximal border of the lateral malleolus |
| 16 | Maximum of concavity along the lateral border of the posterior distal tuberosity |
| 17 | Most medial point of the medial malleolus |
| 18 | Maximum of concavity on the proximal border of the medial malleolus |
| 19 | Maximum of concavity along the medial border of the anterior distal tuberosity |
| 20 | Most anterior point of the anterior distal tuberosity |
| 21 | Maximum of concavity along the lateral border of the anterior distal tuberosity |
| 22 | Maximum of depression on the distal surfaces of the distal epiphysis |
| c0; c1 | Most distal border of the lateral side of the lateral condyle |
| c2; c9 | Outline of the fossa fibularis/insicular tibialis |
| c4; c5 | Proximal and anterior border of the lesser trochanter |
| c6; c7 | Outline of the CFL-BR fossa |
| c8; c9 | Posterior border of the 4^th^ trochanter |
| c10; c11 | Outline of the fibular crest |
| c12; c17 | Outline of the surface of contact with the fibula |
| c18; c28 | Outline of the articular surface of the distal epiphysis |
